# New potential determinants of disability in aged persons with myocardial infarction: results from the KORINNA-study

**DOI:** 10.1186/1471-2318-14-34

**Published:** 2014-03-19

**Authors:** Philip Andrew Quinones, Hildegard Seidl, Rolf Holle, Bernhard Kuch, Christa Meisinger, Matthias Hunger, Inge Kirchberger

**Affiliations:** 1 Institute of Epidemiology II, Helmholtz-Zentrum München, German Research Center for Environmental Health, Neuherberg, Germany; 2 KORA Myocardial Infarction Registry, Central Hospital of Augsburg, Augsburg, Germany; 3 Institute of Health Economics and Health Care Management, Helmholtz-Zentrum München, German Research Center for Environmental Health, Neuherberg, Germany; 4 Department of Internal Medicine/Cardiology, Hospital of Nördlingen, Nördlingen, Germany; 5 Department of Internal Medicine I – Cardiology, Central Hospital of Augsburg, Augsburg, Germany

**Keywords:** Myocardial infarction, Aged, Rehabilitation, Activities of daily living

## Abstract

**Background:**

Elderly individuals with coronary heart disease are a population particularly burdened by disability. However, to date many predictors of disability established in general populations have not been considered in studies examining disability in elderly acute myocardial infarction (AMI) survivors. Our study explores factors associated with the ability to perform basic activities of daily living in elderly patients with AMI.

**Methods:**

Baseline data from 333 AMI-survivors older than 64 years included within the randomized controlled KORINNA-study were utilized to examine disability assessed by the Stanford Health Assessment Questionare Disability Index (HAQ-DI). Numerous potential determinants including demographic characteristics, clinical parameters, co-morbidities, interventions, lifestyle, behavioral and personal factors were measured.

Disability was defined as a HAQ-DI ≥ 0.5. After bi-variate testing the probability of disability was modeled with logistic regression. Missing covariate values were imputed using a Markov Chain Monte Carlo method.

**Results:**

Disability was significantly more frequent in older individuals (Odds Ratio (OR): 1.10, 95% Confidence Interval (CI): 1.05-1.16), patients with deficient nutrition (OR: 3.38, 95% CI: 1.60-7.15), coronary artery bypass graft (CABG) (OR: 3.26, 95% CI: 1.29-8.25), hearing loss in both ears (OR: 2.85, 95% CI: 1.41-5.74), diabetes mellitus (OR: 2.56, 95% CI: 1.39-4.72), and heart failure (OR: 3.32, 95% CI: 1.79-6.16). It was reduced in patients with percutaneous transluminal coronary angioplasty (PTCA) (OR: 0.41, 95% CI: 0.21-0.80) and male sex (OR: 0.48, 95% CI: 0.27-0.85).

**Conclusions:**

Effects of nutrition, hearing loss, and diametrical effects of PTCA and CABG on disability were identified as relevant for examination of causality in longitudinal trials.

**Trial registration:**

ISRCTN02893746.

## Background

Acute myocardial infarction (AMI) and coronary heart disease (CHD) are the most common causes of death and the fourth largest contributor to burden of disease worldwide [1,2]. In 2008/09 22.9% of all Germans aged 65 and older were diagnosed with CHD [3]. The short term lethality of AMI in industrialized countries is in decline [4,5] due to new treatment and prevention procedures [5,6], while the general populations are ageing [7,8]. These developments result in a growing proportion of elderly AMI-survivors. As elderly individuals with chronic diseases are often multi-morbid, they become vulnerable to development of disability, causing increase of costs for care and treatment [8].

Disability in activities of daily living (ADL) indicates the individual’s ability to perform actions fundamental to daily life [9] under the burden of existing health conditions [10]. The ADL concept of disability addresses the individual’s ability to perform self-care and satisfy basic needs such as washing, toileting, dressing, eating, mobility, etc. [11,12]. Reduced ability to perform ADL indicates reduced autonomy and increased dependency on assistance in everyday life [11,12], resulting in need for nursing care and/or other benefits and services. ADL-disability has been shown to be predictive of institutionalization, mortality and healthcare expenditure [13-16].

While heart diseases such as CHD have been associated with disability in older adults in several studies [13,17-21], only few studies to date research disability within the growing population of AMI survivors [18,22,23]. Elderly AMI-survivors in our study were reported to be particularly burdened by prevalent ADL-disability, when compared to the general population of our study region [24]. Indications to whether there are differences in the causation and development of disability between the general population and AMI survivors remain unknown to date. However many factors considered to be associated with or causally related to disability in general or in other populations [8,13,17,18,21-23,25-27], have not been considered in previous studies examining disability in elderly AMI-survivors [18,22,23]. Thus factors affecting the process of disability development in elderly AMI survivors must still be identified and examined. Cross-sectional analyses provide identification of associations relevant for subsequent longitudinal examination of potential causal relationships.

Knowledge of determinants of disability in older aged AMI patients may allow for the development of interventions aimed at preventing or treating disability. Considering the expected increase of this population in the next decades, successful disability prevention and treatment will be a major public health concern.

Thus, the aim of this analysis was to identify clinical parameters, treatment related factors, co-morbidities, socio-demographic and lifestyle factors which show the most substantial association with disability within the population of elderly AMI survivors.

## Methods

### Study sample

The “Koronarinfarkt Nachbehandlung im Alter” (KORINNA) study is a randomized controlled trial with two study arms in which the effect of a nurse-led case management intervention on death and hospital readmission is examined within one year after discharge. Cross-sectional analyses were conducted on the population of the KORINNA-study at randomization. Details on the intervention program, exclusion criteria and the outcome measures were reported elsewhere [28]. The study design was approved by the ethics committee at the Bavarian Chamber of Physicians (Date of approval: 11.11.2008, Reference number: 08064). Furthermore the study was conducted in accordance to German privacy law and in compliance with the Helsinki Declaration. All participants submitted written informed consent before being enrolled in the study. The population of KORINNA consists of 338 home-dwelling patients aged ≥ 65 years who were discharged after treatment of first or recurrent AMI in the central hospital of Augsburg between September 2008 and May 2010 [28]. For the present analysis five patients were excluded due to missing outcome scores.

### Study measures

Most of the data were collected by a study nurse in a face-to-face interview with the patient and a physician during an examination shortly before hospital discharge. In addition, further information, specifically on clinical data, was obtained from information collected on the patients within the MONICA/KORA myocardial infarction registry [29].

### Assessment of ADL-disability

Disability of study participants was assessed with the Stanford health assessment questionnaire disability index (HAQ-DI). The HAQ-DI is one of five health dimensions which constitute the HAQ [30]. It consists of eight domains: dressing and grooming, arising, eating, walking, hygiene, reach, grip, and activities. The applied scoring procedure takes into account items addressing functional ability to perform ADL as well as items assessing utilization of equipment and personal assistance [30]. Numerous studies confirm validity and applicability of the HAQ-DI as a generic instrument [14,30-32]. Disability was defined as a HAQ-DI score higher or equal to 0.5 as suggested by Siegert et al. [33] and applied as a dichotomous cut-point in a cohort study of the general population of elderly community dwelling adults in the Netherlands [8].

### Identification and assessment of associated factors

Based on the results of previous studies [8,13,17,18,21-23,25-27], potentially associated factors were selected from the available data. Chosen variables were assigned to five categories: Demographics, clinical parameters, interventions, co-morbidities, and finally lifestyle, personal & environmental factors (see Tables 1 and 2). Included factors assessed by instrument scores were: (a) social support questionnaire (F-SozU); (b) Mini Mental State Examination (MMSE); (c) Geriatric Depression Scale (GDS); and (d) the abbreviated version of Seniors in the community: risk evaluation for eating and nutrition, version II (SCREENII). Scores were dichotomized according to clinically relevant cut-points developed and confirmed in previous research [34-38]. Infarction type was diagnosed as either AMI with ST-segment elevation in electrocardiogram (STEMI) or AMI without ST-segment elevation (NSTEMI) according to guidelines of the European Society for Cardiology [39]. Blood pressure was categorized according to the American Heart Association, which recommends a blood pressure lower or equal to 130/80 for AMI survivors [40]. Pathological hearing loss in both ears and recent decline in visual acuity were measured according to the geriatric assessment by Lachs et al. [41]. Likewise pathological lung sounds and oedema of lower limb were assessed by the study physician. Obesity was defined as a body mass index of ≥ 30 kg/m^2^[42]. Educational status was defined by the minimal amount of school years necessary to attain the highest school degree held by the participant (9 years/10 years/≥12 years). Heart failure was defined as New York Heart Association classes III and IV diagnosed by physician.

**Table 1:** Age-stratified distribution of population characteristics

	**Population aged 65–74 years n = 152**			**Population aged 75–92 years n = 181**		
**Variable**	**Total %**^**a**^	**No disability %**^**a**^	**Disability %**^**a**^	**Total %**^**a**^	**No disability %**^**a**^	**Disability %**^**a**^
**Demography**						
Male sex	69.7	75.0	60.7	56.4	70.5	49.2
Married	77.5	78.1	76.4	57.6	73.3	49.6
School education	100	36.3	36.7	100	34.7	65.3
≥12 years	14.7	15.8	12.7	4.2	3.45	4.6
10 years	12.7	12.6	12.7	17.4	17.2	17.4
9 years	72.7	71.6	74.6	78.4	79.3	78.0
**Clinical parameters**						
Heart failure	23.0	12.5	41.1	40.9	18.0	52.5
Blood pressure > 130/80 mmHg	17.1	15.6	19.6	23.2	23.0	23.3
Cardiac arrest	13.8	11.5	17.9	17.7	9.8	21.7
Re-infarction	21.7	21.9	21.4	29.4	26.2	31.1
ST-segment elevation	40.1	46.9	28.6	33.2	36.1	31.7
Pathological lung sounds	18.5)	12.6	28.6)	17.9)	8.3	22.7
**Interventions**						
Pacemaker	18.4	9.4	33.9	14.4	6.6	18.3
Coronary artery bypass graft	16.5	7.3	32.1	13.3	6.6	16.7
PTCA^b^ with stent	74.3	87.5	51.8	61.3	75.4	54.2
**Co-morbidities**						
Cognitive impairment^c^	14.1	11.8	17.9	18.5	11.7	22.0
Recent vision decline	23.8	23.2	25.0	30.9	23.2	33.3
Hearing loss both ears	12.2	7.5	20.0	31.2	20.3	36.8
Oedema of lower limb	12.2	5.3	24.1	18.1	6.6	18.1
Diabetes mellitus	33.6	24.0	50.0	32.0	16.4	40.0
Angina pectoris	21.9	20.0	25.0	38.9	30.0	43.3
Circulatory disturbances in legs	12.5	9.38	17.9	23.2	14.8	27.5
Stroke	7.9	5.2	12.5	10.1	5.1	12.6
Osteoporosis	7.2	5.2	10.7	11.1	4.9	14.2
Depressive symptoms^d^	2.8	2.2	3.6	6.3	0.0	9.4
**Lifestyle, personal and environmental factors**						
Social support average or above^e^	93.1	95.6	89.1	80.6	84.8	78.5
Nutrition deficient^f^	81.6	77.2	89.1	82.4	66.1	90.6
Smoker or ex-smoker	64.5	64.6	64.3	52.4	61.0	47.8
Never smoker	35.5	35.4	35.7	47.7	39.0	52.3
Obesity^g^	29.0	28.1	30.4	20.0	11.5	24.2

^a^Percentages of factors with missing values refer to complete cases respectively.

^b^Percutaneous transluminal coronary angioplasty.

^c^Mini Mental State Exam <24.

^d^Geriatric Depression Scale ≤10.

^e^F-SozU <3.29.

^f^Seniors in the Community: risk evaluation for eating and nutrition, Version II <43.

^g^Body Mass Index ≥ 30 kg/m^2^.

**Table 2:** Age- and sex-adjusted logistic regression analyses on prevalent disability (Health Assessment Questionnaire-Disability ≥ 0.5)

**Variable**	**Odds ratio**^**a**^	**95% confidence interval**	**Wald p-value**^**a**^
**Demography**			
Male sex	0.51	[0.31-0.83]	0.006
Married	0.75	[0.43-1.30]	0.300
School education			
≥12 years	ref.	ref.	ref.
10 years	0.99	[0.36-2.77]	0.991
9 years	1.14	[0.49-2.66]	0.771
**Clincial parameters**			
Heart failure	4.37	[2.50-7.64]	<0.001
Blood pressure > 130/80 mmHg	0.89	[0.50-1.59]	0.694
Cardiac arrest	2.18	[1.11-4.28]	0.024
Re-infarction	0.96	[0.56-1.65]	0.876
ST-segment elevation	0.60	[0.37-0.97]	0.037
Pathological lung sounds	2.87	[1.49-5.52]	0.002
**Interventions**			
Pacemaker	4.52	[2.21-9.25]	<0.001
Coronary artery bypass graft	5.77	[2.69-12.38]	<0.001
PTCA^b^ with stent	0.24	[0.14-0.41]	<0.001
**Co-morbidities**			
Cognitive impairment^c^	0.51	[0.27-0.99]	0.045
Recent vision decline	1.12	[0.66-1.89]	0.669
Hearing loss both ears	2.53	[1.35-4.72]	0.004
Oedema of lower limb	4.11	[1.82-9.28]	0.001
Diabetes mellitus	3.23	[1.91-5.47]	<0.001
Angina pectoris	1.60	[0.95-2.68]	0.075
Circulatory disturbances in legs	2.14	[1.13-4.06]	0.020
Stroke	2.39	[0.97-5.90]	0.059
Osteoporosis	2.08	[0.83-5.25]	0.120
Depressive symptoms^d^	0.17	[0.04-0.78]	0.023
**Lifestyle, personal and environmental factors**			
Social support average or above^e^	1.82	[0.87-3.78]	0.110
Nutrition deficient^f^	0.30	[0.15-0.57]	0.001
Smoker or ex-smoker	0.94	[0.57-1.55]	0.796
Never smoker	ref.	ref.	ref.
Obesity^g^	1.38	[0.80-2.36]	0.245

^a^Odds ratios, p-values and confidence limits displayed in Table 2 are products of age- and sex-adjusted, unstratified logistic regression models of the effect of the factors on prevalent disability. The factor sex is adjusted for age only.

^b^Percutaneous transluminal coronary angioplasty.

^c^Mini Mental State Exam <24.

^d^Geriatric Depression Scale ≤10.

^e^F-SozU <3.29.

^f^Seniors in the Community: risk evaluation for eating and nutrition, Version II <43.

^g^Body Mass Index ≥ 30 kg/m^2^.

### Statistical analyses

In initial descriptive analyses frequencies of identified variables were examined within the age groups 65–74 and 75–92. The distributions of age in years and BMI were also examined with histograms. Subsequently all variables were subjected to age- and sex- adjusted, explorative testing against the dichotomized HAQ-DI score with logistic regression. The variable sex was tested in a model only adjusted for age. Variables were subjected to model fitting if significant at a chosen α = 0.05 and smallest cell frequencies proved sufficient for stable multivariate analyses.

The probability of disability was modeled by fitting of a dichotomous logistic regression model by stepwise backward elimination and confirming covariate structure with stepwise forward selection each at α = 0.05. To avoid the exclusion of participants due to missing values in the covariates, five imputed data sets were created using the Markov Chain Monte Carlo method for continuous variables with arbitrary missing data pattern [43,44]. Imputation was not subjected to rounding or setting of interval limits to avoid bias [44]. Model fitting was performed for each imputed data set respectively. Multicollinearity was tested in each imputed data set respectively by assessing variance inflation of the weighted linear model [45]. The continuous variable age was tested for linearity of its association to the logit of the outcome probability using Box-Tidwell tests in both fitted and empty regression models yielding no violation of the linearity assumption. Interactions with sex were tested via interaction terms. Sex-stratified analysis was not performed, since interaction terms showed no significant interactions of sex with any other variables of the finalized model. Finally, results of the fitted logistic regression analysis of the five imputed data sets were subjected to combining analysis to obtain finalized parameter estimates.

All statistical analyses were performed using SAS software, release 9.2 (SAS Institute, Cary, NC).

## Results

According to HAQ-DI categories by Siegert et al. 47.1% persons were self-sufficient, 25.2% had minor difficulties, 16.8% had major difficulties, and 10.8% had a severe handicap in performance of ADL [32,33]. Of 333 patients 52.9% were classified as disabled at hospital discharge. Characteristics of the total sample are displayed stratified according to dichotomized disability status and the age groups 65–75 and 75 and older in Table 1. Disability was less prevalent in the younger age group (36.8%) than in older individuals (66.3%). The sample included more men (62.5%) than women. Males were slightly less common among older (56.4%) than among younger individuals (69.7%). Age of participants ranged from 65 to 92 years with an average of 75.5 years. The majority of the sample (75.7%) had the lowest education level. AMI with ST-segment elevation occurred in 36.3%, and heart failure in 32.7% of the participants. Heart failure and re-infarctions were more frequent among the older (40.9% and 29.4% respectively) when compared to the younger group (23.0% and 21.7% respectively). ST-segment elevation infarctions were slightly less common among older patients (33.2%) than younger (40.1%). The most common co-morbidity was diabetes (32.7%). The rarest health conditions, each of which represented less than 10% of the sample size, were osteoporosis, stroke and moderate to severe depressive symptoms. Among 14 different variables with missing values, school education had the most with 16 missings constituting 4.8% of the complete sample size.

Disabled patients (77 years) significantly differed (p < 0.001) from patients without disability (72 years) in median age. Results of the age- and sex-adjusted explorative analyses are displayed in Table 2. The variable sex was tested in a model only adjusted for age. Sex, heart failure, pathological pulmonary sounds, hearing loss in both ears, oedema of lower limb, diabetes mellitus, circulatory disturbances in legs, pacemaker implant, coronary artery bypass graft (CABG), percutaneous transluminal coronary angioplasty with stent (PTCA), deficient nutrition, cardiac arrest, ST-elevation and cognitive impairment showed significant associations with disability in age- and sex adjusted analyses and were subjected to multivariate analysis (see Table 2). Moderate to severe depressive symptoms were too infrequent for stable multivariate analysis.

The 10 model fitting procedures (forward selection and backward elimination methods in each of the five imputed data sets) produced identical covariate structures. Variance inflation factors within each imputed data set showed values well below 2.5. This delivered no indication of multicollinearity [45].

Results of the finalized regression model for probability of disability are displayed in Table 3. Odds of disability were reduced in males (OR: 0.46, 95% CI: 0.27-0.85) and participants with PTCA (OR: 0.41, 95% CI: 0.21-0.80). The highest odds of disability were found among participants endangered by nutritional deficiency (OR: 3.38, 95% CI: 1.60-7.15), participants who had undergone CABG (OR: 3.26, 95% CI: 1.29-8.25), participants with heart failure (OR: 3.32, 95% CI: 1.79-6.16) and participants with hearing loss in both ears (OR: 2.85, 95% CI: 1.41-5.74). Furthermore the odds of disability increased with each year of rising age (OR: 1.10, 95% CI: 1.05-1.16) and prevalent diabetes mellitus (OR: 2.56, 95% CI: 1.39-4.72).

**Table 3:** Finalized logistic regression model of the probability of disability (Health Assessment Questionnaire-Disability ≥ 0.5)

**Variable**	**β**^**a**^	**Odds ratio**^**b**^	**95% confidence interval**
Intercept	-8.22	0.00	[0.00 - 0.02]
PTCA^c^ with stent	-0.89	0.41	[0.21 - 0.80]
Male sex	-0.73	0.48	[0.27 - 0.85]
Age in years	0.10	1.10	[1.05 - 1.16]
Diabetes mellitus	0.94	2.56	[1.39 - 4.72]
Hearing loss both ears	1.05	2.85	[1.41 - 5.74]
Coronary artery bypass graft	1.18	3.26	[1.29 - 8.25]
Heart failure	1.20	3.32	[1.79 - 6.16]
Nutritional status deficient	1.22	3.38	[1.60 - 7.15]

^a.^All estimates are generated by combining analysis of five imputed data sets.

^b^All odds ratios displayed in Table 3 are products of the fully adjusted model. All factors, for which the model adjusts are compiled in the table.

^c^Percutaneous transluminal coronary angioplasty.

## Discussion

In our present study a reduced odds of disability was associated with male sex and patients who had undergone PTCA. On the other hand we could show that increased odds of disability were associated with rising age, diabetes mellitus, hearing loss in both ears, CABG, heart failure and deficient nutritional status.

Our results confirmed associations of the demographic factors age and sex with disability after AMI demonstrated in previous studies. Studies including AMI-survivors found disability or loss of physical function was more frequent in women and older individuals irrespective of applied measure. Instruments utilized in past studies included the Disability Scale by Rosow and Breslau, the SF-12 and the HAQ-DI [18,22,23]. Studies examining associations between sex, age and ADL-disability in general elderly populations, confirm our results [8,21]. Among these, results from a recent cross sectional study by Strobl et al. seem most comparable with our study, since it utilized data representative of our study region and age group from the KORA-Age cohort and applied the same instrument (HAQ-DI) to measure disability [21]. It found increased odds of disability in women (OR: 2.49, 95% CI: 2.06-3.02) and increasing odds of disability with rising years of age (OR: 1.12, 95% CI: 1.11-1.14). These estimates resemble our findings. However, in contrast to our study Strobl et al. defined prevalent disability as a HAQ-DI score > 0 due to less frequent disability in the general population. Only 22.5% of the general elderly population scored higher than 0.49 (minor to severe disability) in the HAQ-DI. In our population of elderly AMI survivors, 52.9% scored higher than 0.49. Furthermore, proportions of women, who have higher probability of being disabled, are considerably higher in the general population (51.2%) than in AMI survivors (37.5%) [21].

In our study AMI treatments showed strong associations with ADL-disability. The increased odds of disability in patients with CABG may be related to the severity of the intervention itself, which initially affects the patients’ general health status negatively. As this study examined patients shortly after the AMI event, it cannot be excluded that CABG will prevent disability in the long term. In contrast, PTCA as a more gently reperfusion intervention was shown to be associated with a reduced odds of disability already at the time of hospital discharge. On the long term, however, Dodson et al. have not found any significant effect of PTCA on disability (measured by EuroQol-5D questionnaire) one year post-AMI in 2002 patients with AMI with a mean age of 59 years [22].

Heart failure emerged as the strongest cardiac indicator of prevalent ADL-disability. Previous research discovered a decline in ability to perform ADL among individuals with heart failure during the first year after AMI [18]. The long-term prediction of disability assessed with patients from the Framingham disability study, identified heart insufficiency to be predictive of disability in women [17]. However, when interpreting the above it must be considered that among the discussed studies none applied the HAQ-DI to assess disability.

Our finding that AMI patients with diabetes were more likely to be disabled is in line with results of studies on the older aged general population. For instance, Gregg et al. found that diabetes was associated with a 2- to 3-fold increased risk of physical disability, accessed by physical performance tests and self-rated ability to walk ¼ mile, climb 10 steps and do housework, in a sample of 6,588 U.S. adults aged 60 years or older [46].

The strong associations of hearing loss in both ears and nutritional status are noteworthy as they have not been taken into account in available studies examining disability within the population of AMI patients. In studies on the general elderly population, hearing impairment was found to be a significant predictor of disability [9,47]. Strobl et al. investigated determinants of disability within the framework of KORA-Age and found sufficiently nourished individuals, as defined by the SCREEN malnutrition score, to have significantly reduced odds of any ADL-disability as assessed by the HAQ-DI (OR: 0.93, 95% CI: 0.92-1.56). However, as discussed above, since Strobl et al. defined prevalent disability as a HAQ-DI score > 0 due to much more infrequent disability in the general population, coefficients of the regression models are not directly comparable with our present study [21]. Furthermore, a recent review of literature on the association between nutrition and mobility in elderly people suggested that low micronutrients correlate with mobility disability [48]. While this study applies measures so different that comparability to our study remains questionable, the congruence of laboratory-based and questionnaire-based results remains noteworthy. These findings suggest a further examination of potential causal effects nutritional deficiency and hearing loss may have on disability in elderly AMI-survivors. Furthermore adjustment for these factors may be necessary to avoid confounding in future studies.

Sample characteristics and associations found in the bi-variate analysis are mostly confirmatory of observations made in past studies [21-23,18,13,17]. A few non-significant findings of the bi-variate baseline analysis may well have clinical relevance. For instance, participants with STEMI showed slightly reduced odds of disability. One possible interpretation is the fact that patients with STEMI may be diagnosed earlier and this leads to earlier access to necessary clinical interventions which reduce further damage of the heart muscle and consequently may prevent disability.

### Strengths and limitations

To our knowledge, this study is the first which explores factors associated with disability in patients with AMI aged 65 or older. A large number of different variables including clinical characteristics and cardiac treatment could be tested for their association with disability not considered in any of the related studies to date. As part of the randomized KORINNA-study, instruments were carefully selected and standardized assessments were performed. The covariate structure of the finalized regression model was confirmed to be a very stable model of the most dominant influence factors on the outcome in different selection procedures.

Findings relevant to future research on disability in AMI-survivors were made. Due to the large effect sizes it appears reasonable that adjustment for hearing loss and nutritional deficiency may be relevant to the estimation of other predictors in clinical research. Finally, our study raises the question whether differences in disability prevalence between elderly AMI-survivors and other populations are systematic or arbitrary. If disability differentials are caused by random differences in certain population characteristics such as hearing loss, adjustment is sufficient. If however, the structure in causal relationships between factors predictive of disability differs from other populations, the development of a disease-specific theoretical framework will be essential.

However, some methodological limitations should be considered. Since the analysis was conducted with data from a study not originally designed for this research question, sample size did not allow consideration of potentially relevant factors too infrequent (n < 40) for the execution of a stable multivariate analysis. Examples are the effects of social support and moderate to severe depressive symptoms on disability found in the bi-variate analyses. Furthermore, the exclusion of relevant characteristics with too low frequencies resulted in the unfeasibility of structural equation modeling. Thus, potentially relevant causal relationships between covariates could not be considered. In addition, effects may be overestimated. The ratio of predictors over outcome events in the full model is 1/11.2. Application of the rule of thumb proposed by Steyerberg and Harrell, suggests that with a ratio over 1/10 shrinkage while advisable is not absolutely necessary [49]. Finally, due to the cross sectional design, actual causations between disability and covariates cannot be proven in this study.

## Conclusions

Our study has confirmed that factors which are found to be associated with disability in populations other than AMI patients, also account for disability development in AMI patients aged 65 years and older. Since nutrition and hearing loss were identified as most important associations with disability, they should be examined in subsequent longitudinal studies. If causality is shown they should be adjusted for in future studies examining determinants and treatment of disability in AMI survivors. Beside demographic characteristics and co-morbidities, different reperfusion treatments strongly and diametrically influenced the occurrence of disability at hospital discharge. Further studies are needed to clarify whether these effects persist over time.

## Competing interests

The authors declare that they have no competing interests.

## Authors’ contributions

PAQ developed the study question, performed all data analyses and drafted the manuscript. HS prepared the data sets performed plausibility checks and variable transformations. CM, IK, RH and HS developed the complete KORINNA study design and data assessment. MH was advisor for statistical methods. IK was general counselor and advisor. All authors reviewed and revised preliminary manuscript drafts and approved the final manuscript.

## Pre-publication history

The pre-publication history for this paper can be accessed here:

http://www.biomedcentral.com/1471-2318/14/34/prepub
